# The many modes of flagellar and ciliary beating: Insights from a physical analysis

**DOI:** 10.1002/cm.21656

**Published:** 2021-03-15

**Authors:** Charles B. Lindemann, Kathleen A. Lesich

**Affiliations:** ^1^ Department of Biological Sciences Oakland University Rochester Michigan USA

**Keywords:** axoneme, central pair, doublets, dynein, sperm

## Abstract

The mechanism that allows the axoneme of eukaryotic cilia and flagella to produce both helical and planar beating is an enduring puzzle. The nine outer doublets of eukaryotic cilia and flagella are arranged in a circle. Therefore, each doublet pair with its associated dynein motors, should produce torque to bend the flagellum in a different direction. Sequential activation of each doublet pair should, therefore result in a helical bending wave. In reality, most cilia and flagella have a well‐defined bending plane and many exhibit an almost perfectly flat (planar) beating pattern. In this analysis we examine the physics that governs flagellar bending, and arrive at two distinct possibilities that could explain the mechanism of planar beating. Of these, the mechanism with the best observational support is that the flagellum behaves as two ribbons of doublets interacting with a central partition. We also examine the physics of torsion in flagella and conclude that torsion could play a role in transitioning from a planar to a helical beating modality in long flagella. Lastly, we suggest some tests that would provide theoretical and/or experimental evaluation of our proposals.

## 1 INTRODUCTION

The eukaryotic flagellum/cilium, also called an undulipodium, is one of the most versatile cellular organelles for generating useful work. They propel the sperm of most metazoans and are used in feeding and reproduction of protists and for moving fluids in complex multicellular organisms. To fill such a range of useful applications they must convert the force generating action of thousands of molecular motors, in this case the AAA motor, dynein, into macroscopic bending of a much larger complex structure, the microtubular axoneme.

**FIGURE 1:**
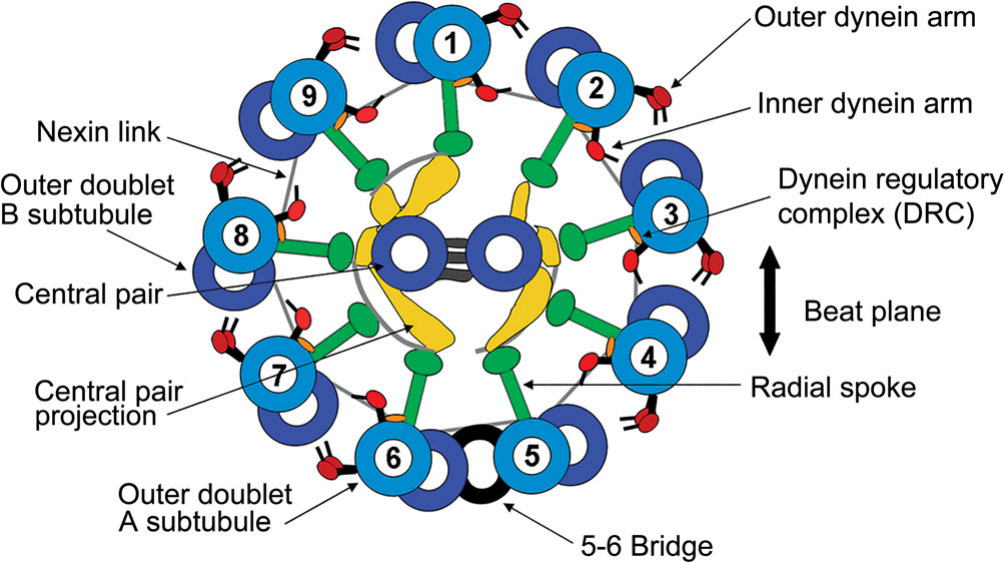
The eukaryotic flagellar axoneme. A schematic diagram showing the component structures in a typical axoneme of a cilium or flagellum. The dynein arms located on the outer doublet microtubules are minus end directed motor proteins that generate force to bend the flagellum. The plane defined by the central pair of single microtubules is typically perpendicular to the primary beat plane of the flagellum or cilium as indicated by the large arrows. Reproduced with permission from Lindemann and Lesich (2010) [Color figure can be viewed at wileyonlinelibrary.com]

The axoneme, which is a scaffold of microtubules and associated proteins, is the structural basis of undulipodia. It is highly conserved throughout hundreds of millions of years of evolutionary descent. As illustrated in cross section in Figure 1, it consists of nine doublet microtubules arranged in a circle around a central apparatus consisting of two microtubules, referred to as the central pair (CP), and a series of associated protein projections. The outer nine doublets are evenly spaced around this central apparatus by nine spoke‐like constructions termed the radial spokes. These spokes contain many structural proteins as well as enzymes and signal receptors. The ring of nine doublets is stabilized by flexible linkages between the outer doublets themselves (Warner, 1976). These linkages were originally called the nexin links. More recent research defines them as the dynein regulatory complex (DRC). Overall, this basic, yet complex, scaffold is often referred to as the 9 + 2 axoneme, and retains much the same general design across a wide spectrum of eukaryotic life forms.

The force‐generating motor proteins that power axoneme movement are located on the outer doublets in a pattern that repeats at 96 nm intervals. They are arranged in two rows as seen in Figure 1. The dyneins are oriented such that they project from each doublet toward the next doublet and, if the flagellum is viewed from the base toward the tip, all point in a clockwise direction. The dynein motor proteins generate the motive force to bend the flagellum and cause it to move with a whip‐like motion through the surrounding fluid. This motion is referred to as the flagellar or ciliary beat. It is known that most flagella and cilia beat with the major axis of bending perpendicular to the axis defined by the two microtubules of the CP. When no CP is present the beat is most often helical, although there are exceptions (Idei et al., 2013; Mitchell, 2017). Because the circle consists of nine elements, there are more doublets on one side of this axis than on the other. It has become standard nomenclature to label the single doublet that occupies the middle position on the side with fewer doublets as Doublet # 1 and to number the others, in order, in a clockwise direction with the dynein also pointed clockwise, as shown in Figure 1.

Dynein is a minus‐end directed motor; it pushes the doublet, on which it permanently resides, toward the flagellar base. This is because the microtubule doublets assemble from the base, making the base the minus end of the tubule. Thus, each doublet can be viewed as the cargo of its own array of motors and is carried base‐ward when those motors are active. The stalk of each dynein heavy chain has a microtubule binding domain that can attach to the adjacent doublet. Force is produced when the heavy chain changes conformation and transmits strain through its stalk to the neighboring doublet. Sliding occurs between the doublets when the stalks of the dyneins translocate stepwise along the neighboring doublet. When dyneins are active between a pair of neighboring doublets, the doublet on which the dyneins are permanently bound is pushed base‐ward while the adjacent doublet experiences an equal and opposite force in the tip‐ward direction.

This presents a somewhat amusing scenario to anyone attempting to intuit how the flagellum works. If all of the dyneins around the circle of nine are simultaneously active, it is akin to a circle of nine people each attempting to lift his or her neighbor up. Assuming they all are of equal strength; it is safe to say that no one gets lifted. The upward and downward forces on each will cancel and there will be no net force and ultimately no motion. This is equally true in the axoneme. Accordingly, something must break the circle of activity or a flagellum could never produce a beat.

Experimental evidence tells us that most cilia and flagella beat with a strong preference to bending in the direction perpendicular to the CP, which is the plane defined by Doublets #1 and #5–6. We must be content with saying most, because in some cilia and flagella the CP rotate. Yet, even in those instances, the principal beating plane is usually aligned with the axis defined by the plane of a line extending from the center of Doublet #1 to between Doublets #5 and #6. The most notable example of this is in *Chlamydomonas*, a green alga which possess the most studied flagella in the world. This is the same principal beat direction as in cilia and flagella that have a fixed position of the CP. The metazoa, to which all multicellular animals belong, follow the fixed CP pattern. The fact that the primary beating plane is the same regardless of whether the CP rotates or not would suggest that some other factor, beside the orientation of the CP, must be responsible for breaking the symmetry of the ring and causing the motors to bend the structure in a preferred direction.

**FIGURE 2:**
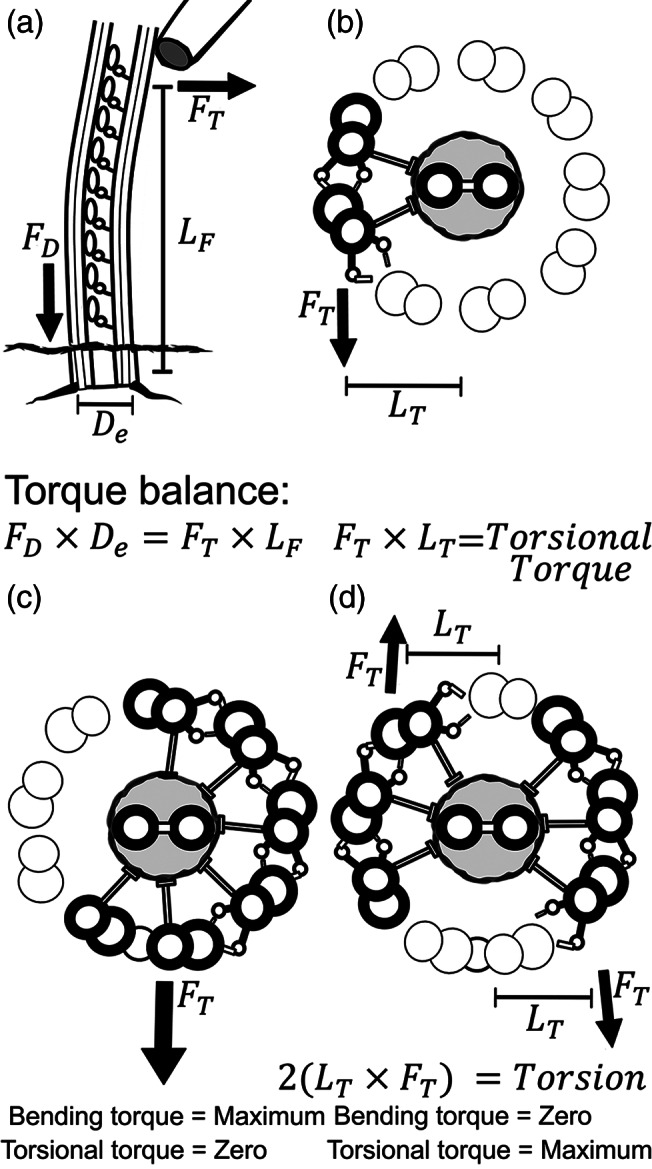
Bending torque and torsion in flagellar mechanics. (a). Activation of the dynein motors between a single pair of doublets produces a tension/compression force couplet (*F*_D_), which acts on the lever arm of the doublet spacing (*D*_e_). If bending of the flagellum is prevented by an external probe (or other resistance), the resulting torque balance is the lever arm from the anchored base to the position of the probe (*L*_F_) multiplied by the resulting force (*F*_T_). The vector direction of the applied bending torque is defined by the center to center axis of the active doublet pair as shown in (b). This relationship allows us to find the lateral force (*F*_T_), if we know the dynein force. The lateral force (*F*_T_) acting on displacement of the doublet pair from the central axis of the flagellar axoneme yields the maximal torsional torque that can be delivered to the flagellar structure at the point of resistance. (c) If all the doublet pairs are active between Doublet #1 and #5–6 the bending torque is increased by the increased effective diameter, but the torsional component becomes zero as the force axis is aligned with the center of the axoneme. (d) In contrast, if multiple doublets are active off axis and on opposite sides of the axoneme, both active groups contribute torsional torque. While the bending torque from dynein motors on the opposite sides of the axoneme oppose each other, the torsional components are of the same chirality, so they are additive. This condition would maximize torsion in any regions of the flagella that have overlapping areas of dynein activation on both sides of the axoneme

If the dyneins on only one doublet were activated, it would push the doublet with the active dyneins toward the flagellar base and the doublet the dyneins are walking along will be pushed toward the flagellar tip, as illustrated in Figure 2. An active doublet pair is the simplest way the dynein motors could generate the force and torque to bend the flagellum, and the simplest regulatory scheme is to activate only the dyneins on one doublet at a time. This would allow the production of a force couple on just two doublets at a time, one being pushed toward the base and the next one being pushed toward the tip. The physical spacing of the doublets allows the accumulated tension on one and compression on the other to generate a bending torque. This torque acts to bend the structure. The torque generated by such an arrangement is aligned parallel to the axis of separation of the two doublets, as in Figure 2b. Each doublet pair has a unique center to center axis of separation. Therefore, if the dyneins on each of the doublets were activated sequentially around the circle of the axoneme, it would bend the flagellum in a three‐dimensional wave that resembles a helix.

The initial discovery of CP rotation in ciliates (Omoto & Kung, 1979, 1980) and then in *Chlamydomonas* (Kamiya, 1982; Omoto & Witman, 1981) suggested that such a scheme might be feasible. The rotating CP might act as a rotor that could activate selected dyneins via contact with the radial spokes. Additional functional support for this hypothesis was provided by the work of Smith and Sale (1992a) who showed that the radial spokes could modulate inter‐doublet sliding. This is still debated as a possibility, at least in the cilia and flagella that have a rotating CP (Smith & Yang, 2004; Wargo, McPeek, & Smith, 2004). Many cilia, including those of paramecium and most protist ciliates, have a three‐dimensional beat pattern that is somewhat conical, like a truncated helix (Holwill & Satir, 1990; Sugino & Machemer, 1987, 1988; Sugino & Naitoh, 1983). Bull sperm flagella also exhibit a beat that has a three‐dimensional component that takes the form of a flattened helix.

Rikmenspoel (1965) first quantitated the three‐dimensional movement of the bull sperm flagellum and showed that the minor axis of the helical flagellar wave is about one third the amplitude of the major axis. Subsequent studies on other mammalian sperm have confirmed that they also exhibit a three‐dimensional beat to varying degrees (Vernon & Woolley, 1999; Woolley, 1977; D. M. Woolley & Osborn, 1984).

The conversion of the force generated by the dynein motors of cilia or flagella into a helical bending of the axoneme is what one would expect with a circular arrangement of the doublets. It is also quite easy to explain a flattened helical beat, as there are structures in the axoneme of many cilia and flagella that provide additional stiffness in one plane. An immobile, non‐rotating, CP and permanent bridges between Doublets #5 and #6 are common features of metazoan cilia and flagella. These structural elements make the axoneme stiffer in the axis parallel to the CP microtubules, and would be expected to decrease the bend amplitude in the plane of the CP, effectively flattening the helix. What is much more difficult to understand is how the 9 + 2 axoneme can, in many instances, produce a beat that is very nearly planar. That is the one of the issues we address in this report.

### 1.1 Flattening the circle

Early electron micrographic studies of the axoneme by pioneers such as Björn Afzelius, Ian Gibbons and Don W. Fawcett (B. Afzelius, 1959; B. A. Afzelius, 1961; Fawcett, 1958; I. R. Gibbons, 1961b) provided much of the flagellar axoneme's structural details. Gibbons (1961a) was the first to make the correlation between the principal beating plane of cilia and flagella and the orientation of the CP microtubules. These studies also revealed that two of the outer doublets, #5 and #6, are often bridged to each other by permanent connections. These two doublets, if they were to generate force between them would tend to supply torque to bend the axoneme perpendicular to the major beating plane. Conversely, permanently bridging them prevents them from sliding relative to each other and, therefore, makes the axoneme stiffer and harder to bend in the axis parallel to these doublets. Ishijima, Sekiguchi, and Hiramoto (1988) showed that in horseshoe crabs, sperm from the American species, which have a CP, exhibit a planar beat, whereas the Asian species, which lack a CP, show a more helical beating pattern. This suggests that the CP plays a role in generating the flat beat pattern. Eel sperm is another example of a switch to a helical beat associated with the lack of a CP (B. H. Gibbons, Baccetti, & Gibbons, 1985).

**FIGURE 3:**
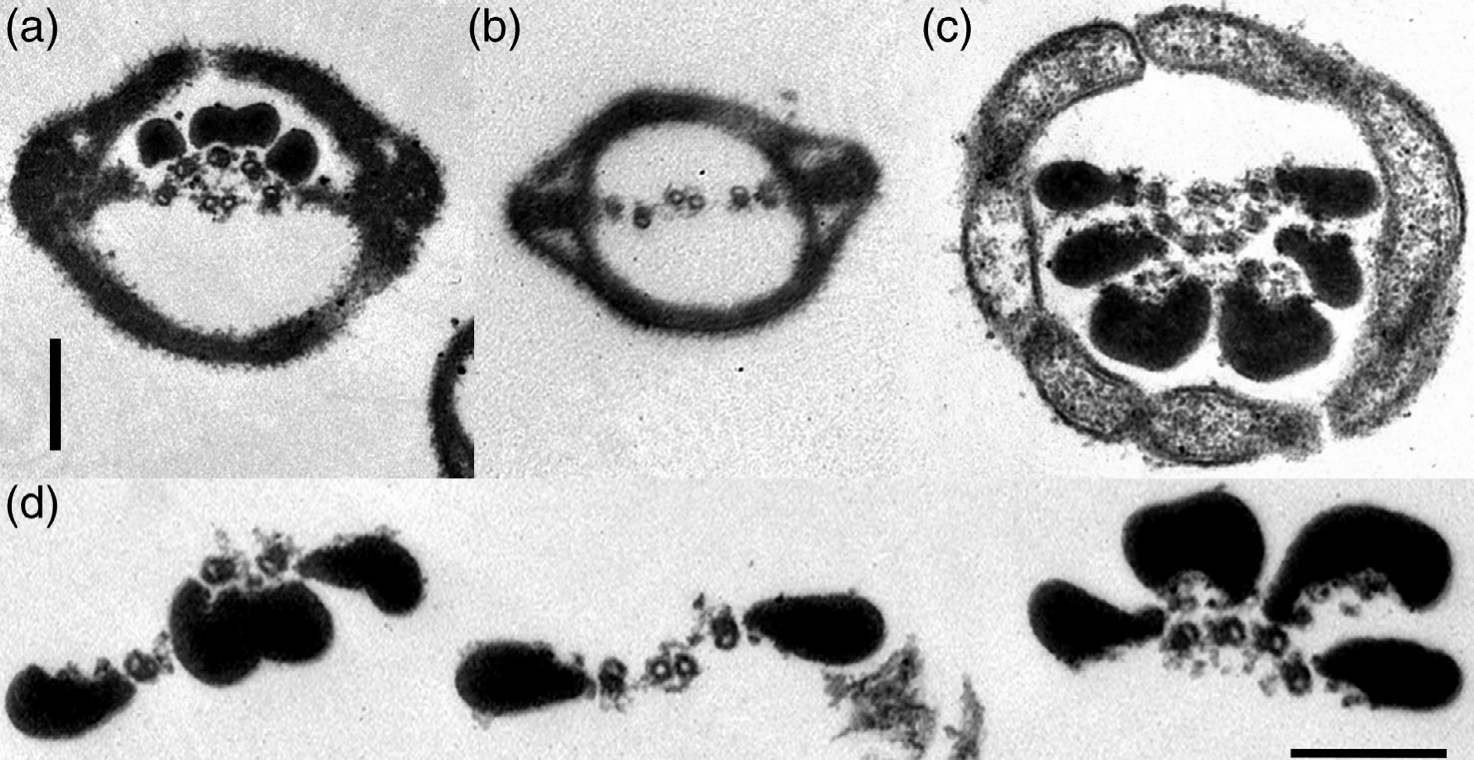
Transmission electron micrographs of partitions in rat sperm. A collage of micrographs showing characteristic disintegration fragments from rat sperm flagella after activating interdoublet sliding by Mg‐ATP. Central partitions composed of the #3‐central pair and #8 are present in all four images. Crosssections (a) and (c) are missing the #4, 5, 6, 7 and the #9,1,2 group, respectively. Both groups are missing in (b), leaving only the 3‐CP‐8 partition. (d) Shows a middle piece disintegrated into all three groupings. The figure is composed from micrographs produced using the method in Lindemann et al. (1992). Bars indicate 200 nm

Our own studies of sliding disintegration in rat and bull sperm flagella demonstrated that, in addition to not rotating, the CP apparatus appears to be permanently connected to the spokes of Doublets #3 and #8, in what we described as a partition (Lindemann, Orlando, & Kanous, 1992). Figure 3 shows electron micrographs of rat sperm flagella that disintegrated by inter‐doublet sliding and exhibit intact #3‐CP‐8 partitions. A similar attachment of the CP to certain spokes has been observed and reported in sea urchin sperm flagella (Shingyoji & Takahashi, 1995). Therefore, it appears that in metazoan cilia and flagella, where the CP do not appear to rotate, the reason they do not is because there are structural attachments that stabilize the orientation of the CP. Bridging the CP apparatus to Doublets #3 and #8, and possibly also Doublet #7 in sea urchin (Shingyoji & Takahashi, 1995), will resist shear displacement between these doublets. This structural feature makes the axoneme more resistant to bending in the plane aligned with the CP. Therefore, both the #5–6 bridges and the #3‐CP‐8 partition act to stiffen the axoneme in the same plane.

**FIGURE 4:**
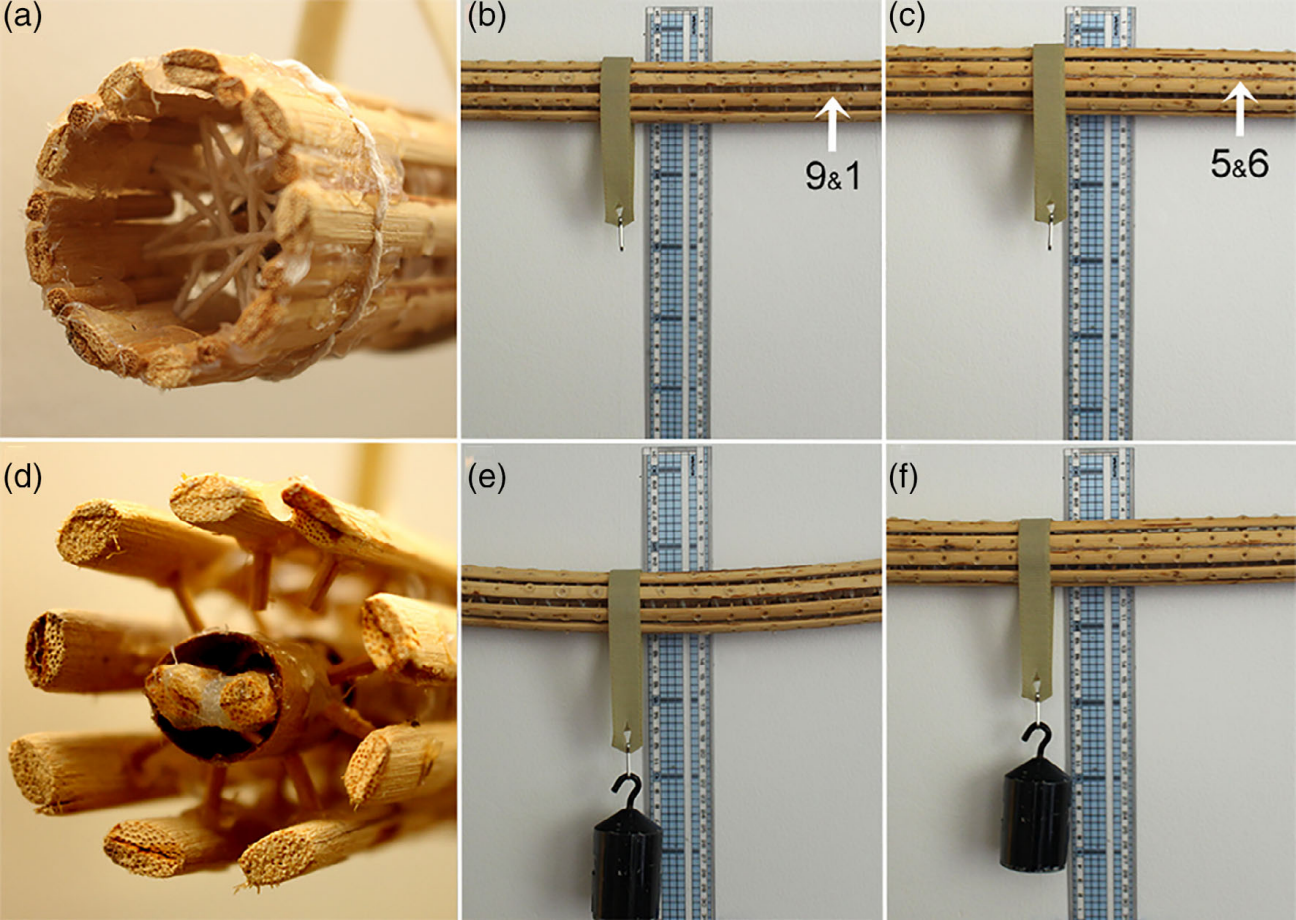
The effect of the axoneme structures on stiffness. A wooden model of a 9 + 2 axoneme was constructed from basket weaving staves and small dowels. Like a real flagellar axoneme, the model possesses a basal anchor similar to a basal body, shown in (a). Elements representing Doublet #5–6 are permanently linked with silicone adhesive, shown in (c). The central pair (CP) of elements are surrounded by sections of cylindrical cardboard sheath that are linked by silicone adhesive to the spokes of elements representing Doublets #3 and #8, visible in (d). This forms a central partition similar to that observed in most flagella. The model is flexible allowing measurement of the relative resistance to bending in the axis parallel to the CP and perpendicular to the CP, as shown in (b–e) and (f). The structure was 2.6 times stiffer in the axis parallel to the CP demonstrating that these structural features impart a significantly different bending resistance in the normal beat plane of a flagellum as compared to the off‐axis plane. Reproduced with permission from Lindemann and Lesich (2016) [Color figure can be viewed at wileyonlinelibrary.com]

Under this circumstance, torque produced by dyneins on the doublet pairs that are aligned out of the principal bending plane, such as those on Doublets #9 and #1, will have a reduced effect simply because the axoneme is more resistant to bending in the direction of the applied torque. Exactly how much the stiffness of the passive (non‐motile) axonemal scaffold varies in the two bending directions has not yet been experimentally determined. We estimated the effect of these structural features by assembling a flexible model made from wooden reeds and silicone adhesive. This construction is shown in Figure 4. The model has a CP, radial spokes, and permanent linkages of the #3 and #8 spokes to the central apparatus. It also has the #5 and #6 outer elements permanently connected to each other. We measured the stiffness of this model and found that the ratio of stiffness in the axis parallel to the CP was 2.6 times the stiffness in the axis perpendicular to the CP (Lindemann & Lesich, 2016).

The relative geometric spacing of the 9 + 2 reed elements of the model were taken from an electron micrograph of a *Tetrahymena* cilium. Thus, the geometry should be a fairly good representation of the relative spacing of structural elements in a real axoneme. There is some uncertainty in the estimate, deriving from the unknown flexibility of the #3‐CP‐8 partition elements. The spokes are a composite structure of many proteins and the separation between Doublets #3 and #8 is quite large (180 nm). Therefore, it is likely that it may be more flexible in a real cilium than in the wooden model. If so, our estimate of the off‐axis stiffness is likely to be on the high side. Nonetheless, it is the best estimate available until a direct measurement of bending resistance in the two directions of a real cilium is accomplished experimentally.

Given that an axoneme with these features is on the order of two times as stiff in the direction aligned with the CP axis, one would expect the beat to be biased in the more flexible axis perpendicular to the CP. That is often what is seen in the beating of both cilia and flagella. It is quite sufficient to explain the flattened helical beat of bull sperm flagella as documented by Rikmenspoel (1965) where the beat amplitude in the minor axis is about 1/3 of the major axis. It is not sufficient, however, to explain the second observation made in that same study which showed a proportion of the sperm, under the same conditions, exhibit an almost completely flat beat. Such observations of alternate beating patterns are common not only in mammalian sperm but also in flagella of widely different species (C. J. Brokaw, 1966, 1975, 1996; Holwill & McGregor, 1976; Koyfman et al., 2011; Omoto & Brokaw, 1985; D. M. Woolley, 2007; D. M. Woolley & Vernon, 1999, 2001).

The flagellar beat of some sea urchin species is exceptionally flat. This was documented in studies by Shingyoji et al. (Shingyoji, Gibbons, Murakami, & Takahashi, 1991; Shingyoji, Katada, Takahashi, & Gibbons, 1991) working in the lab of Ian Gibbons. When sperm were held by the head with a suction pipette and rotated so as to align the beat with the line of sight, the flagellum appeared to be motionless. While this may be an extreme example, very planar beating is not uncommon in sperm flagella of invertebrates and is also observed in some vertebrate sperm as well.

We must also consider that Brokaw (1966, 1975) as well as Woolley and Vernon (2001) showed that elevated viscosity, a purely external mechanical factor, can convert the beating pattern of sea urchin sperm to a helical configuration. In addition, Ishijima (2012) showed that mechanical constraint can have a similar effect on converting the beat pattern from planar to helical in tunicate and sea urchin sperm. These observations demonstrate conclusively that the same underlying axoneme can produce both a helical or a planar beat depending on loading conditions. Sea urchins, a metazoan organism, have a fixed CP and permanent #5–6 bridges so these observations must somehow be reconciled with this structural limitation as well.

Equally remarkable is that the beating pattern of a *Chlamydomonas* flagellum can be almost as flat during the organism's dominant forward swimming pattern. This is difficult to understand, as *Chlamydomonas* does have a rotating CP and lacks a permanent #5–6 bridge. Although there is no single obvious structural bias that could be called upon to make the flagellum stiffer in one axis and define a preferential beating plane, there is an accumulated body of evidence for asymmetry within the *Chlamydomonas* flagellum (Bui, Sakakibara, Movassagh, Oiwa, & Ishikawa, 2009; Dutcher, 2020).

What does a *Chlamydomonas* flagellum possess which breaks the circle and defines a beating plane? Unlike the other doublets, in *Chlamydomonas*, Doublet #1 is missing the outer arm row of dyneins. In addition, there is evidence of a bridge present between Doublets 1 and 2 (Bui et al., 2009; Hoops & Witman, 1983; Lin, Heuser, Song, Fu, & Nicastro, 2012) but it is sporadic, as it is observed in only an average of 30% of the images (range=21‐38%). These linkages, as first described by Hoops and Witman (1983), may play a role somewhat similar to the 5–6 bridges in metazoan flagella. This means the torque produced to bend the flagellum in the off‐axis is decreased as a result of the #1–2 pair acting as a weak link in the circle. This reduces the amount of bending torque transmitted across the axoneme to bend the flagellum in the non‐preferred bending plane, but only in one direction of the beat, since there is no comparable structural anomaly on the opposite side.

These observations tell us some things that are quite difficult to reconcile. The sea urchin beat tells us that even if the flagellum has a preferred axis of flexibility, it is not sufficient to explain a perfectly flat beat. A ratio of 2.6–1 is only sufficient to reduce the helical component of the bending wave, not eliminate it. Likewise, bull sperm cannot change to a flat beat from a helical beat just based on differential stiffness, nor can differential stiffness explain how sea urchin sperm change from planar to helical beating at elevated viscosity. *Chlamydomonas*, having only the sporadically observed 1–2 doublet linkages to provide additional stiffness in the out of plane direction, cannot be subject to the same mechanical constraints as sea urchin or bull sperm. Yet, they too, can exhibit a planar beating mode. This leaves us with the following question: how is it possible that the same circular arrangement of motors can produce both a helical beat or a nearly flat beat?

### 1.2 The physics of flagellar bending

**FIGURE 5:**
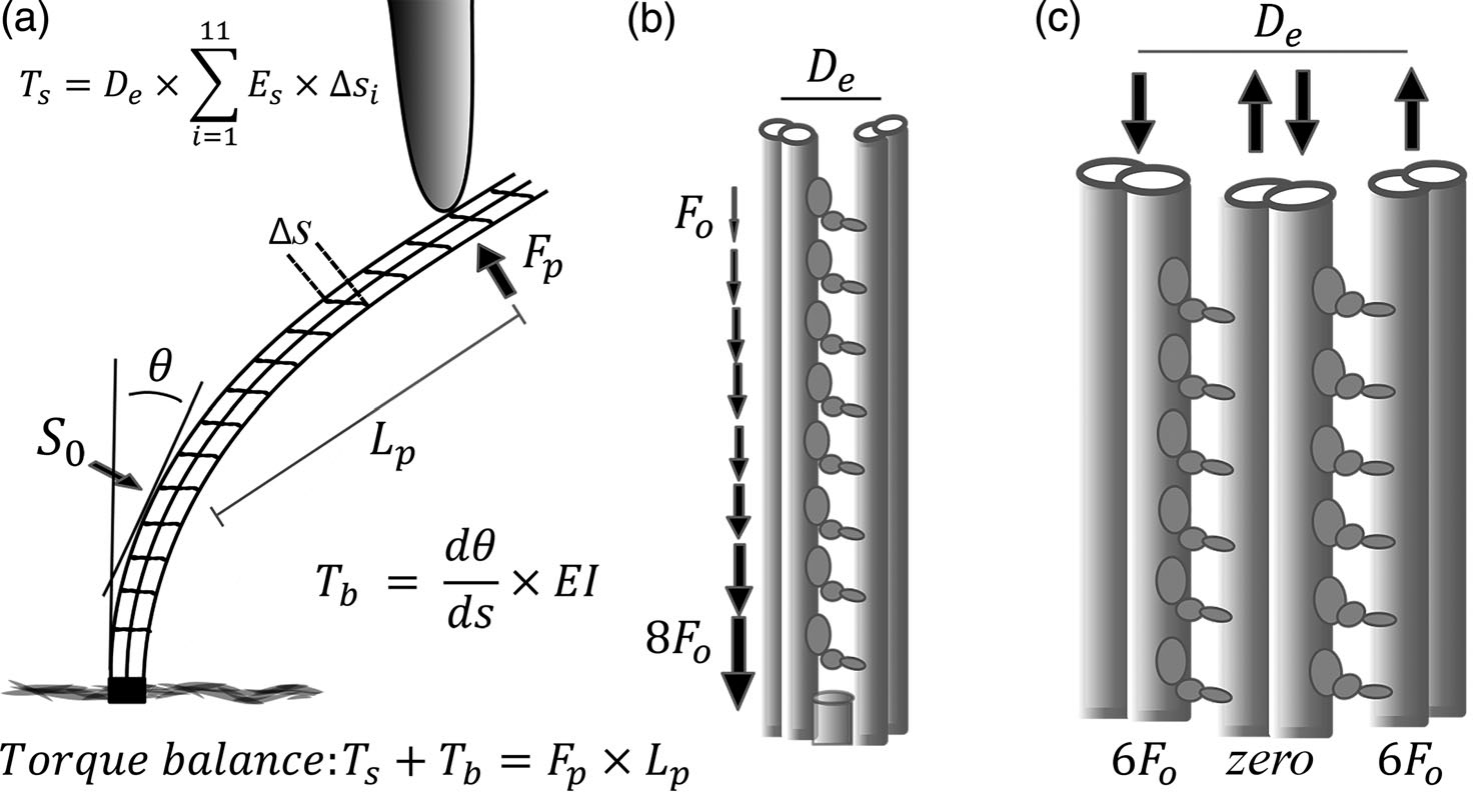
An analysis of passive and active bending in a flagellum. (a). Passively applied bending torque from an external probe. The force applied by the probe is opposed by an equal and opposite resistive force (*F*_p_), which is the sum of the resistance to the doublets bending and the resistance of the inter‐doublet linkages (DRC/nexin) to stretching. The curvature, *dθ*/*ds*, at any given point (S_0_), multiplied by the structural resistance to bending (EI), yields the bending torque (*T*_b_). The shear between the doublets at each interdoublet linker (Δ*s*) times the elastic resistance to shear (*E*_s_) of the linker contributes a tension and compression on the doublets which acts upon their separation (*D*_o_) to create additional torque which also resists bending (*T*_s_). The sum of the bending and shearing torque (*T*_s_) is balanced against the externally applied torque (*L*_p_ × *F*_p_). (b). Bending torque from internal motors. The active dynein motors between a single pair of doublets each contribute a small increment of force (*F*_o_) which adds to the total force accumulated at the basal anchor (flagellar basal body). The torque generated to bend the flagellum is the accumulated dynein force at each position along the flagellum multiplied by the lever arm that is the center to center spacing of the doublets which is the effective diameter (*D*_e_). (c). When dyneins on a series of adjacent doublets are activated in unison, the doublets between the first element in the series and the last element in the series experience no net tension or compression, as shown. The total tension and compression on the end elements are the same as in the case of a single doublet pair, but the effective diameter (*D*_e_), is doubled and hence the bending torque, which is the product of the tension and effective diameter, doubles as well. For simplicity, only the active forces from dynein are considered in diagrams (b) and (c). The force and torque contributions to bending resistance from inter‐doublet linkages are presented in (a)

The first principle in flagellar (and ciliary) mechanics is the requirement for the application of torque, not just force, to bend a slender elastic structure. Dynein motors can develop motive force, but the force by itself is not sufficient to cause a cilium to bend unless that force is applied to a lever arm. Force applied to a lever arm is called torque and it has the units of force × distance. When the flagellum is bent with a probe, the probe exerts an external force on the flagellum. The bend induced at each position along the flagellum must be balanced by the applied torque supplied by the probe. The applied torque at each position is proportional to the distance of that position from the externally applied force, multiplied by the magnitude of the applied force in the direction perpendicular to the axis of the lever arm. This principle is illustrated in Figure 5a. The elastic rigidity, or stiffness, of the flagellum at each point is balanced against this applied torque at each and every point along the flagellum. In a passive flagellum that is bent by a probe, and is stationary after being bent, the physical principle that applies is a simple Newtonian equilibrium between the applied torque at each position and the elastic resistance of the structure at that same position. This is a simple and straight‐forward situation to understand.

When the torque to bend the flagellum does not come from an external application of force, but from the internal rows of dynein motors, the force accumulates on the outer doublets with each of the motors contributing a small share. The dynein heavy chains are attached to one doublet and form transient attachments to the neighboring doublet by way of a projection called the stalk. When the dynein heavy chain molecule changes its configuration in response to ATP hydrolysis, a force is exerted between the adjacent doublets. Because the dynein molecule spans a gap between the two adjacent doublets, the dynein arm complex itself must also bear a locally acting torque as defined by the lever arm of the separation and the tangentially directed force applied to the doublets. This issue has been presented and considered in other analyses (Hu & Bayly, 2018; Lindemann & Hunt, 2003). For this analysis we need only consider the tension and compression contributed by the dyneins to the doublets.

The doublets are essentially anchored at the basal body, thus the tension or compression from all of the active dyneins accumulates at that location. At each position further from the base, only the dyneins distal to that point are contributing to the tension or compression acting at that position. This is also illustrated by the size of the force vector arrows in Figure 5b. The tension on the doublet being pulled away from the base is always exactly balanced by the compression on the adjacent doublet being pushed toward the base. This is because they are generated by the same subset of dynein motors pushing one doublet with the same force as they are pulling the other doublet. This tension/compression couplet is acting across the lever arm formed by the separation of the two doublets. The bending torque at each position is the result of this force couple acting over the lever arm of the doublet separation.

Since the dyneins are spaced evenly along the doublets and the separation between the doublets is relatively uniform, the bending torque generated will be greatest near the basal anchor and will decline linearly as a function of the distance from the base. This is, of course, assuming that all the dyneins along the whole length are active and contributing force.

Others (Bayly & Dutcher, 2016; C. J. Brokaw, 2014; Hu & Bayly, 2018) have hypothesized that instability related to compression of the doublets may underlie the initiation of waveform generation. Above a critical load, resistance to bending can vanish leading to buckling instability. Models have shown that this can be a factor in initiating bending, but are beyond the scope of this analysis.

The resistance to bending in a real flagellum is not uniform due to another anatomical feature of the axoneme that is the inter‐doublet linkages, formerly known as the nexin links. They were identified via cryo‐electron tomography to be part of the complex of proteins called the DRC (Heuser, Raytchev, Krell, Porter, & Nicastro, 2009). These linkages which are spaced at 96 nm intervals along the length of the axoneme, hold the ring of doublets together and resist the sliding of one doublet along the next. This resistive element has been demonstrated in sea urchin, rat, bull, and mouse sperm flagella and most recently in *Chlamydomonas* flagella (Lindemann, Macauley, & Lesich, 2005; Minoura, Yagi, & Kamiya, 1999; Pelle, Brokaw, Lesich, & Lindemann, 2009; Xu et al., 2016). It is likely a universal feature of the axoneme. This resistance is not a product of the dynein motor attachments as it is still present when the dyneins have been deactivated by vanadate (Lindemann et al., 2005; Pelle et al., 2009), or by high ATP (Minoura et al., 1999), or are partially absent due to mutations (Xu et al., 2016). The best evidence suggests it is a product of the inter‐doublet linkages present in the DRC complex. The resistive action of these linkages results in a counterbend in the distal flagellum when the proximal flagellum is bent with a probe.

Analysis of the counterbend effect in sea urchin has shown that this shear resistance can contribute significantly to the overall bending resistance, and when shear between the doublets is large, it can account for more than half of the bending resistance (Pelle et al., 2009; Xu et al., 2016). Consequently, the final shape that is induced by the action of the dynein motors is also determined in part by this shear resistance component. Brokaw was first to realize this factor in the behavior of the axoneme and incorporated separate components of bending resistance into his early models of the sliding filament mechanism. It is noteworthy that he did this work long before shear resistance was shown to exist experimentally (C. J. Brokaw, 1972a, 1972b).

Following Brokaw's lead, when the geometric clutch computer model was developed (Lindemann, 1994a, 1994b) these two separate sources of bending resistance were incorporated into the model. Continuous activation of all the dyneins on one side of the flagellum in the computer model resulted in fish hook‐ or candy cane‐like configurations (Lindemann, 1994b, 1996). This is significant because many flagella and cilia assume this shape in the presence of high levels of free calcium ion (C. J. Brokaw, Josslin, & Bobrow, 1974; B. H. Gibbons & Gibbons, 1980; Lindemann & Goltz, 1988; Moritz, Schmitz, & Lindemann, 2001; P. Satir, 1975; P. Satir, Reed, & Wolf, 1976). Calcium ion (Ca^2+^) is known to alter the beat symmetry of cilia and flagella in all organisms where it has been studied. Apparently, in most cilia and flagella, Ca^+2^ has the effect of favoring the activation of the dyneins on one side of the axoneme selectively (Lesich et al., 2012; Lesich, dePinho, Dionne, & Lindemann, 2014). A surplus of the ion seems to lock those dyneins “on” continuously. It is therefore likely the calcium arrest phenomenon exhibited by many cilia and flagella is showing us the equilibrium state that results when the dyneins that bend the axoneme in one direction are activated and reach a static balance against the total bending resistance of the axoneme.

In any situation where the flagellum or cilium is arrested and not moving through the surrounding fluid, is similar to the situation where the flagellum is bent by external application of force. The only difference is that the simple Newtonian balance is between the dynein generated torque at each position along the flagellum and the total bending resistance at the same location. This relationship was recognized long ago by Machin (1958, 1963) and can be expressed at every position as:

(1) $$M_{\text{active}}+M_{\text{elastic}}=0$$

Of course, flagella are designed to move and when they do, they must push the surrounding fluid. This creates a drag on the movement (viscous drag) which exerts an external force on the flagellum and is the normal mode of flagellar or ciliary operation. Viscous drag resists the action of the active torque generated by the dyneins as well. The result is a third source of torque that must be included in the Newtonian balance. Machin (1958, 1963) recognized this component, including it in the full expression of the torque balance:

(2) $$M_{\text{active}}+M_{\text{elastic}}+M_{\text{viscous}}=0$$

Note that in the equation there is no consideration of mass or acceleration because at the scale of a flagellum in viscous fluid these influences are negligible.

### 1.3 What determines the beat plane?

Equation (2) is conceptually the most important relationship for us to understand how the beat is accomplished and how it is shaped. In a working flagellum or cilium, it is the action of dynein that generates the active moment of torque (*M*_active_). This active torque bends the flagellum and is resisted by the other two sources of torque. If the flagellum is not impeded by the proximity of a surface or a strong viscosity gradient, the drag resistance is essentially uniform in any bending direction. This component of torque depends on the velocity of movement, but is similar in all bending directions. The bending resistance is influenced by the axoneme structure and can be considerably greater, by at least a factor of two, in the plane of Doublets #5–6. As mentioned earlier, this is because a great many cilia and flagella have permanent structural connections between Doublets #5–6 and also between Doublets #3 and #8 and the CP.

The main determinant of bending direction is the axis of the applied active torque. Every doublet pair, with the exception of #5–6 in metazoa, is presumably capable of generating an active bending torque. We can easily see that the torque from each of the eight active pairs is generated in a different direction. If each doublet pair is activated in succession, the direction of bending torque applied to the flagellum would rotate around the circle. Given that the local resistance to bending is not uniform, and may vary by as much as 2.6 greater stiffness in the plane of the 5–6 axis, then the applied active torque is expected to produce only about 1/3 as much bending when the active torque is coming from double pairs #9 and #1 or #1 and #2. Ultimately, the anisotropic nature of the bending resistance can be partially responsible for defining a preferred bending plane. This suggests that purely structural considerations are sufficient to flatten the beat to a helical beat with approximately a 3:1 ratio of amplitude in the helical wave in the preferred axis of flexibility, which coincides with the plane perpendicular to the CP or Doublets #5 and #6.

This is not a globally applicable solution, as many organisms have flagella and cilia without these structural adaptations. The best studied example is the flagella of *Chlamydomonas* which is known to have a rotating CP and does not appear to have permanent linkages between Doublets #5 and #6. In spite of this, *Chlamydomonas* flagella do have a well‐defined beat plane, which is almost entirely planar when they move in the forward swimming direction. It is likely that the absence of the outer row of dynein arms on Doublet #1 in *Chlamydomonas* weakens, or may even prohibit, the transfer of bending torque from Doublets #8 and #9 to the #2 and #3 doublets on the opposite side. This would greatly reduce the bending torque that can develop across the axoneme in the axis perpendicular to the bending plane.

Unfortunately, this would only flatten the beat during the phase where doublets on the #7–8–9 side of the axoneme are active. There is no such anomaly preventing torque from Doublets #4–5–6 and 7 acting together to generate torque that would bend the axoneme in the plane defined by the #5–6 doublets. Some other regulatory mechanism must be preventing the development of torque from Doublets #3 to #8 in these types of flagella.

### 1.4 The flat beat considered

Two physical issues must be considered if we are to understand how a flat beat can be achieved by the flagellar axoneme; the axis of the bending torque and the issue of torsion. There is an interesting feature of the mechanism that governs conversion of dynein force to bending torque that may render somewhat of a solution. If only the dyneins on one doublet are active, the plane of the resulting bending torque will be defined by the center to center separation of the two doublets involved. However, it is much more likely that more than one set of doublets must act together to generate sufficient bending torque to bend the whole axoneme. There is some experimental support for this assumption. When we measured the stalling force of the bull sperm flagellum (Schmitz, Holcomb‐Wygle, Oberski, & Lindemann, 2000) we discovered that it would require the action of all of the dyneins on one side of the flagellum acting together across a lever arm equal to the full axoneme diameter to explain the amount of bending torque that we measured. Even using the full axoneme diameter, the contribution per dynein heavy chain was ~5pN, which is at the extreme upper end of force measurements on isolated dynein (Shingyoji, Higuchi, Yoshimura, Katayama, & Yanagida, 1998). If the bending torque originated from a single doublet pair, then the force per dynein would need to be about four times greater. This is well beyond any direct measurement of dynein force to date and seems to suggest that when a flagellum is actively being bent by the action of the dynein motors, it must be dependent on several pairs of doublets acting in coordination.

As is illustrated in Figure 5c, when more than one pair of adjacent doublets contributes bending torque, the resulting tension is on the end two elements of the active group. The doublets in between experience distal pull from one neighbor and base‐ward push from the other neighbor and hence, the distal and base‐ward forces cancel. Consequently, the resulting tension and compression is transferred to the elements at the ends of the active group. As a result, the axis of the bending torque is defined by the centers of the first and last doublets in the active series, as shown in Figure 5c.

This raises an interesting possibility. If the activation scheme for the flagellar beat activates all of the dynein on the doublets on one side of the axoneme at one time and none on the other side, then all of the torque will be generated between Doublets #1 and #5–6. This defines a plane that is almost exactly orthogonal to the plane of the CP. What could serve to activate all of the dyneins on one side and then all of the dyneins on the opposite side? That is a bit more difficult question to answer. However, the inescapable conclusion is that the physics of axonemal bending requires the application of bending torque along this central axis in order to explain the extremely flat beating plane of flagella such as those of sea urchin sperm.

**FIGURE 6:**
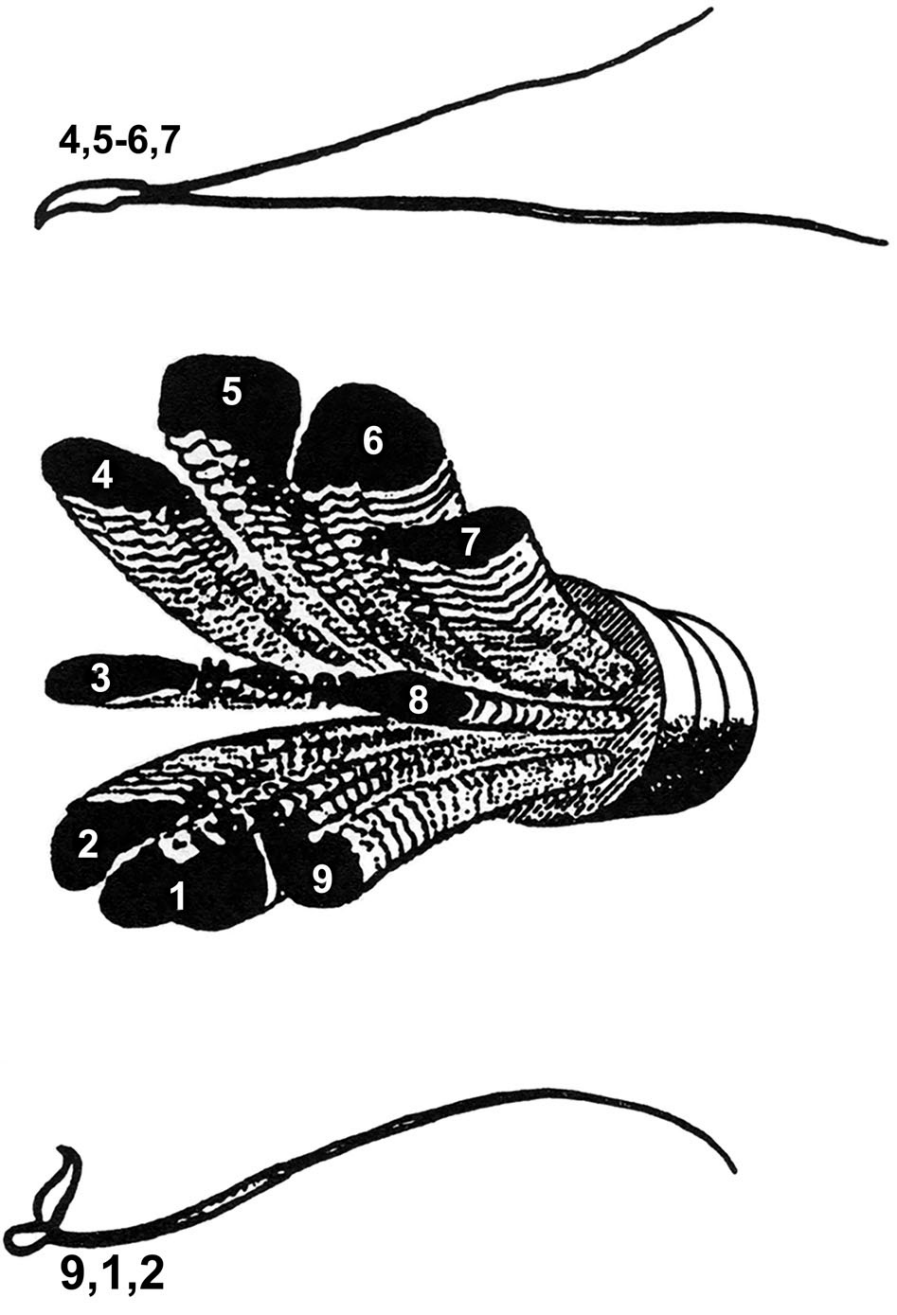
Schematic of the rat sperm partition and doublet ribbons. The drawing summarizes the grouping of elements observed in sliding disintegration experiments on rat sperm. The central pair often remains connected to Doublets #3 and #8 forming a partition of the axoneme. The remaining doublets, with their associated outer dense fibers, often initially slide out of the fibrous sheath as a group before separating into individual doublets. This gives the axoneme the functional equivalent of a central partition and two ribbons of doublets. Reproduced from Lindemann et al. (1992) with permission

**FIGURE 7:**
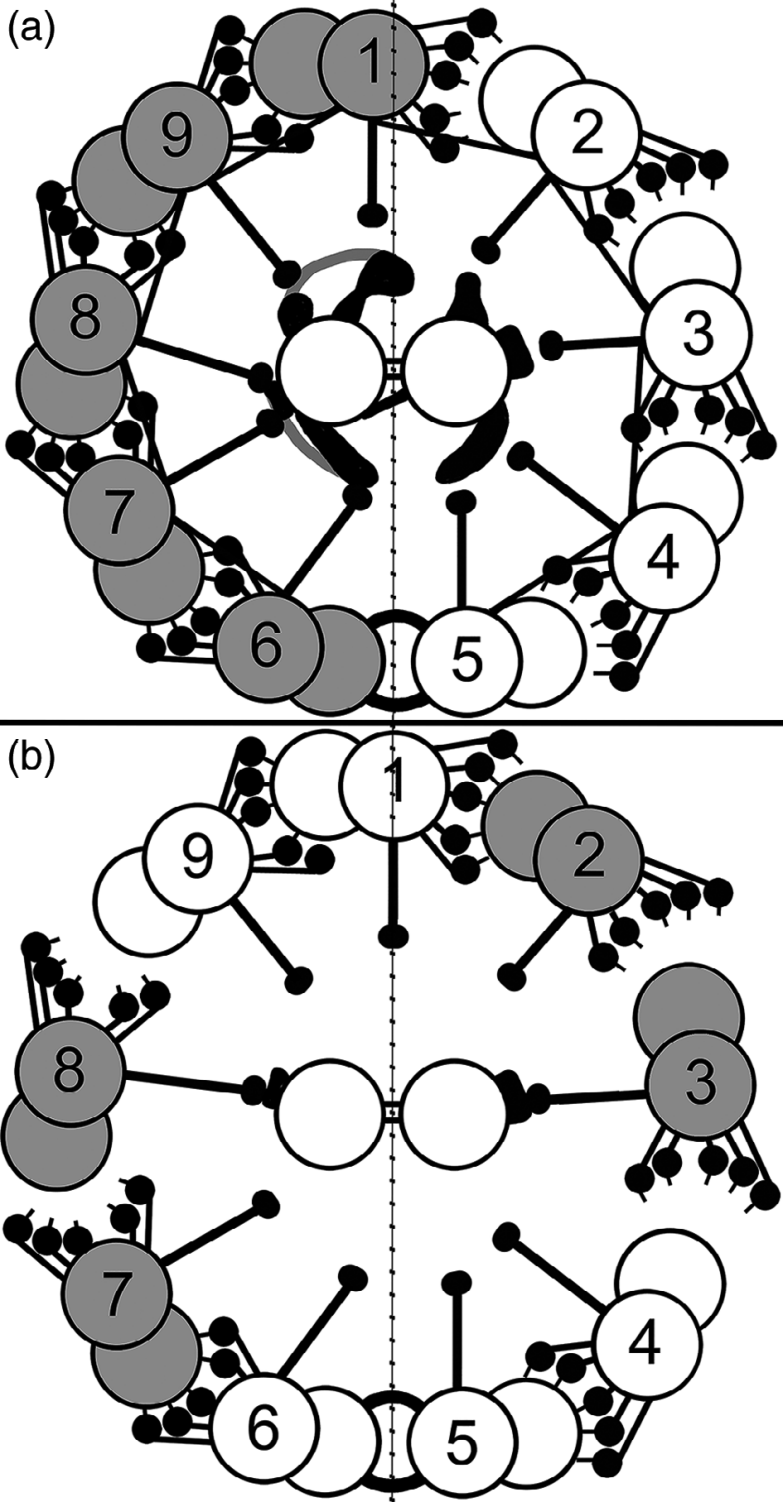
Two possibilities to achieve a planar beat. (a). Co‐activation hypothesis. Simultaneous activation of all of the doublet pairs on one side (darkened in figure), followed by simultaneous activation of all doublet pairs on the opposite side. This would result in all bending torque coming from tension and compression couplets on Doublets #1 and #5–6, which align with the central axis of the axoneme. (b). Ribbon hypothesis. Doublets #9, 1 and 2 slide together as a group, and Doublets #4, 5–6, 7 slide together as a group, while the active force for bend development comes primarily from the dynein on Doublets #7 and 8 on one side and Doublets #2 and 3 on the opposite side. The dyneins on the other doublets are attached but relatively inactive. This arrangement would also produce bending torque aligned with the central axis perpendicular to the central pair (CP). Both of these possibilities would result in very little off‐axis bending and very little torsional torque

Although the simultaneous activation of the dyneins on all of the doublets from one side of the axoneme, followed by the simultaneous activation of the opposite side is one viable possibility, there is another possibility to consider. From experiments which initiated the sliding disintegration of mussel gill cilia (P. Satir & Matsuoka, 1989), rat sperm (Lindemann et al., 1992), bull sperm (Bird, Hard, Kanous, & Lindemann, 1996) and mouse sperm flagella (Kanous, Casey, & Lindemann, 1993; Lesich et al., 2010) by Mg‐ATP, it was shown that bundles of doublets emerge initially instead of individual doublets emerging sequentially one upon the next, and only later do the bundles break apart into individual doublets. These groupings are seen in the micrographs of Figure 3. The doublets that drive the bundles out first are usually, although not always, the doublets that interact with the #3‐CP‐8 partition. Shingyoji and Takahashi (1995), reported somewhat similar findings with sea urchin sperm flagella. This suggested to us that most of the dynein activity bending the flagella is originating from four doublets: # 2, #4, #7 and #9. The remaining doublets are able to disintegrate by sliding but seem, at least at first, to hold the emerging bundles together as a unit. This is schematically illustrated in Figure 6. If, in fact, the axoneme can act as three ribbons, rather than eight or nine independent elements, then this could also result in a very flat beat. Based on the geometry of the axoneme, the two mobile ribbons do ~80% of the sliding during the normal beat cycle. The center of the #9–1–2 ribbon and the center of the #4–5–6–7 ribbon are located directly across the axoneme from each other in a plane perpendicular to the #5–6 axis. Therefore, tension and compression on the ribbons would exert bending torque in line with the axis of Doublets #1 to #5–6. Both of these possible mechanisms to produce a very flat beat are illustrated in Figure 7.

To date, the best view ever achieved of an axoneme frozen while in an active beat was accomplished in the lab of Daniela Nicastro using cryo‐electron tomography (J. Lin & Nicastro, 2018). The work was done on sea urchin sperm, which have a remarkably flat beat. What their results suggest is that most dyneins are potentially active, in a pre‐powerstroke state, but are not cycling through active power strokes that create sliding. It is only when the dyneins on one side of the axoneme enter an inhibited, low‐affinity, state allowing them to slide backward, do the dyneins on the opposite side have the freedom to actively translocate and create bending. It is primarily, but not exclusively, the dyneins which interact with the 3‐CP‐8 partition that undergo an alternation of active inhibition. They report there is some variation between the principal and reverse bends in the number of doublets exhibiting inhibition of dynein with Doublet #4 also involved in the principal bends. Participation of the inner and outer dyneins is also not identical in the two bending directions.

Their interpretation is that all the dyneins in the pre‐activation state are producing force rather than just mechanically anchoring the doublets together. However, one must consider that very little sliding displacement will actually occur between doublets 9–1–2 and doublets 4–5–6–7 in the course of a flat beat oriented perpendicular to the CP. Therefore, it is likely that the dyneins on these doublets, while potentially active, may not be cycling (stepping). If they are not dynamically active, but physically bound to the adjacent doublet in a high affinity state, the resulting picture is most consistent with our three‐ribbon conception in Figure 7b. If all of the non‐inhibited dyneins are actively pulling, it is a better fit with the transfer of tension and compression mainly to doublets 1 and 5–6 as shown in Figure 7a.

### 1.5 The issue of torsion

Another consideration that needs to be mentioned and included in any complete analysis of flagellar movement is the potential for torsion of the axoneme to also be a factor. While most flagella seem to have a well‐defined bending plane which extends along their entire length, activation of any pair or group of doublets that create an off‐center axis of torque will inevitably produce a component of torque which acts to twist the flagellum along its linear axis. Since every pair of doublets is offset from the center of the axoneme by 80–90 nm, activation of any single doublet pair would therefore produce a twisting torque as well as a bending torque. Figure 2 illustrates the origin of this torsional component of torque.

There is some experimental evidence to suggest this may be a contributing factor in the beating pattern of long flagella. It was reported that in hamster sperm (Woolley, 1977; D. M. Woolley & Osborn, 1984; D. M. Woolley & Vernon, 1999) each bend is fairly planar, but there appears to be torsion between alternating bends that results in an overall beating pattern that is three dimensional. These flagella are very long, and each bend entrains the action of many more dynein than act to bend a short cilium. Woolley (2007) also reported that the very long sperm of quail can assume a twisted configuration suggesting internal torsion, and eel sperm lacking the CP also seem to exhibit torsion between planar bends described as helicoid beating (B. H. Gibbons et al., 1985). These observations tend to support the idea that in long flagella, where the torque is produced by a very large number of dynein acting in series, torsion can become a significant factor in the dynamic behavior of the flagellum.

What determines the magnitude of torsional torque, and how does it relate to bending torque? The lever arm for the application of torsional torque to the axoneme is the lateral displacement of the axis of applied bending torque from the central mechanical axis of the intact axoneme, as illustrated in Figure 2b. Consequently, a twisting torque will develop any time bending torque is not aligned with the central axis of the flagellum. As Ishijima (2019) showed in his recent analysis of helical beating, a helical beat requires the sequential activation of doublet pairs. This would be a circumstance ideal for development of torsional force as the bending torque will be continuously off center and be contributed by different doublet pairs, unlike the situation we just considered that is required for planar beating. Twisting torque will therefore develop whenever the condition for planar beating is not met.

If we consider the simplest case, which is a single pair of doublets interacting to produce bending torque, we can begin to understand the physics involved in torsion. The dynein motors between the two doublets contribute the tension and compression necessary to provide torque to bend the flagellum just as we considered above. However, since the direction of the applied bending torque is not aligned to the central axis of the flagellum, some of the force exerted on the flagellum by the action of the dyneins is acting on the lever arm defined by the off‐center distance of the doublet pair from the center of the axoneme. It then becomes a matter of determining the magnitude of that force component to evaluate the torsional torque.

If we consider the balance of forces in a static condition where the flagellum is stationary, then the torque balance involved in torsion may be more easily solved. We conducted a series of experiments where we stalled bull sperm flagella against a force calibrated glass microprobe (Schmitz et al., 2000). When the flagellum was stalled in a relatively straight configuration pushing against the probe, all of the dynein force was balanced by the resistance of the probe and, consequently, the measured force is the stalling force of the dyneins located between the flagellar base and the probe position, as illustrated in Figure 2a. Dyneins beyond that point are free to bend the flagellum and therefore the bending torque they generate is resisted by the bending resistance (stiffness) of the flagellum itself. Under these conditions, the dynein motors in the stalled basal region were measured to produce ~5pN per dynein head (Schmitz et al., 2000). Since this was measured when the motor proteins were stalled, it likely represents the maximum force per dynein.

Based on this estimate, if the ~150 dyneins present along a 1 μm length of doublet are activated, it will produce a net force on a pair of doublets amounting to a tension of 750 pN on one relative to the other. Since the center to center doublet spacing is ~60 nm this yields a bending torque of 45,000 pN·nm or 4.5^e‐17^ N·m. Two doublets within a 20 μm length of flagellum that is securely anchored at the base, would therefore be capable of producing 9^e‐16^ N·m of torque against an external probe. With a 20 μm lever arm, this produces a force of 4.5^e‐11^ N or 45 pN. This pushing force, if it is generated by a doublet pair on one side of the axoneme, as illustrated in Figure 2b, will also act on a lever arm offset from the center by 90 nm. Therefore, it will contribute a torsional torque of 4,000 pN·nm which is ~1/10th of the bending torque.

The physics of the torsional torque has an interesting twist (pun intended). If two doublet pairs on opposite sides of the same axoneme are both contributing force, they will oppose each other by contributing bending torque in opposite directions. However, they will contribute torsional torque in the same twisting direction, as indicated in Figure 2d. This is an odd twist in the story. Every pair of doublets can contribute torsional torque in the same direction! Accordingly, if the dyneins on four doublets pairs are pulling along a 20 μm length of flagellum, 16,000 pN·nm of torsional torque is created. Naturally, a much longer flagellum could also entrain the force from a larger number of dyneins and will have regions of active dynein on both sides of the axoneme, especially when more than one bending wave is present. It is quite likely that in very long flagella, such as a rat sperm or a quail sperm, the torsional torque can be as much as 10 times greater than in our simple example. In these very long flagella, the twisting torque may be of the same general magnitude as the bending torque. Consequently, twisting will be a proportionately greater factor in the beating pattern.

Naturally, both the torsional resistance and the bending resistance will determine how the flagellum reacts to the total dynein force vector. For example, if the flagellum is very easy to bend but difficult to twist, bending will be the dominant response. In contrast, if the torsional resistance is small, twisting will be greater. Torsional resistance has an inverse proportion to length, as each unit of length added makes the total resistance to torsion less in the ratio of *R*/*L*, where *R* is the torsional resistance per unit length and *L* is the length over which the torque acts. This also contributes to making a longer flagellum more susceptible to twisting. As a consequence, increased flagellar length increases the force available for twisting and reduces the resistance to twisting.

## 2 DISCUSSION

The physics of bending an elastic beam by the application of torque requires that the vector direction of the applied torque dictates the direction of bending. In order for a flagellum or cilium to exhibit a very flat beat confined to a specific plane, it follows that the vector direction of the applied force must be closely aligned to the beat plane. This can only be accomplished if the couplet of tension and compression which generates the bending is applied in the axis defined by the centers of Doublets #1 and #5–6. This is dictated by the physical properties of the system and all proposed explanations of how a flat beat can be generated must be consistent with this requirement.

We are not the first to attempt to explain the mechanism that allows flagella to beat with a flat planar beat and to also convert to helical beating. Brokaw (1966, 1975) first noted that sea urchin and other invertebrate sperm could change from a planar to a helical beating pattern in response to external viscosity. This is a very important clue to the underlying mechanism of regulation of the beating pattern. It demonstrates that viscous loading, which is a purely mechanical restraint, can convert the mechanism from one form to the other. Woolley and Vernon (2001) did perhaps the most detailed analysis of the motion of sea urchin sperm in each mode of beating induced by varying the viscosity. They showed that the helical beat was a true propagating and propulsive wave. They also ventured to propose a mechanism for the transition. They hypothesized that the transition could be accomplished only if the order of activation of the doublets changed under increased mechanical load. While their study was not the first to document the ability of the same flagella to switch beating patterns in response to viscosity, it did provide much useful information about the two beating patterns and some thoughtful insights as to the possible mechanism. One particularly interesting suggestion was that helical beating may require simultaneous activation of dyneins on both sides of the axoneme. Interestingly, this would increase the twisting torque component as we have shown.

More recently, Ishijima (2019) did a very extensive analysis of the shear between the outer doublets in a flat beat and a helical beat and from this showed graphically that different specific regions of dynein would have to be sequentially activated to account for the two beating patterns. His analysis is valid and his illustrations help in visualizing the dynamics of the flagellar components that must accompany the two forms of beating. In particular, the shear patterns provide a clue to the sequence of dynein activity involved in the two forms. From our present consideration of the physics of axonemal bending we can contribute some additional insight.

For the planar beat, it could result from full activation of the dyneins on Doublets #6, 7, 8 and 9, alternating with full activation of the dyneins on Doublets #1, 2, 3 and 4. This would transfer all of the accumulated tension first to Doublet #1 and all of the compression to Doublets #5–6, followed by an exact reversal with tension being transferred to #5–6 and compression to Doublet #1. This possibility is schematically illustrated in Figure 7a.

The second possibility is that the axoneme can sometimes behave as two ribbons consisting of Doublets #9–1–2 on one side of the axoneme and #4–5–6–7 on the opposite side. The torque would develop by the action of the dyneins on only Doublets #2 and #3 on one side of the axoneme and those of Doublets #7 and #8 on the opposing side. The result would be a torque with an orientation directed from the centroid of one ribbon to the centroid of the other, which also results in a torque roughly aligned with Doublets #1 and #5–6. This is schematically illustrated in Figure 7b. This scenario is experimentally consistent with several published studies of sliding disintegration in demembranated and weakened intact flagella (Bird et al., 1996; Kanous et al., 1993; Lindemann et al., 1992; Lindemann & Gibbons, 1975; Shingyoji & Takahashi, 1995).

If this second alternative is the correct mechanism to explain how a flat beat can be achieved, then it may also provide some insight into how it is possible for the same flagella to assume different beat characteristics depending on physiological signaling from the cell. It is well documented that many organisms can exhibit both helical and planar beating depending on both external conditions and physiological changes in the state of the organism (C. J. Brokaw, 1966, 1975; S. Ishijima, 2012; Rikmenspoel, 1965; S. S. Suarez, Dai, DeMott, Redfern, & Mirando, 1992; S. S. Suarez, Drost, Redfern, & Gottlieb, 1990; D. M. Woolley, 2007; D. M. Woolley & Vernon, 2001). In his early studies of the free‐swimming motion of bull sperm, Rikmenspoel (1965) documented that most beat with a helical flagellar wave with a major and minor axis of amplitude. The major axis aligns with the plane of the flat disc shaped head. This is consistent with the major axis being aligned with the #1 to 5–6 axis and perpendicular to the CP, which in bull sperm does not rotate. He also noted that a small percentage of the swimming sperm swam in circles and had a flat flagellar beat. We now know from many detailed studies that mammalian sperm switch their beating pattern as they are affected by chemical signals in the female reproductive tract. For more information, see reviews by Freitas, Vijayaraghavan, and Fardilha (2016), and Suarez (2008, 2016), respectively.

How might such versatility be accomplished? The mechanical intactness of the #9–1–2 and #4–5–6–7 ribbons is dependent on the activity state of the dyneins of Doublets #9 and #1 and also of Doublets #4 and #6. If these dyneins are inactive, but attached to the neighboring doublet in the high affinity state that precludes or at least minimizes interdoublet sliding, then the #9–1–2 and #4–5–6–7 groups will slide together as units. However, if instead these same dyneins located on Doublets #9, #1, # 4, and #6 were activated, it would convert a flat beat into a helical beat.

This naturally raises the question: what could be acting to repress the action of those dyneins? We propose that the radial spokes, in coordination with the DRC is in control of this function. There is a great deal of accumulated evidence that the radial spokes and the CP projections play a role in governing motility. This subject is too vast to be considered here, but has been reviewed elsewhere (B. H. Gibbons et al., 1985; Pigino & Ishikawa, 2012; Smith & Yang, 2004; Teves, Nagarkatti‐Gude, Zhang, & Strauss, 2016; Zhu, Liu, & Yang, 2017).

Smith and Sale (1992a, 1992b) showed that dynein mediated sliding is inhibited when doublets lack radial spokes. Therefore, this natural inhibition must be released in order to allow dyneins to participate in torque production. If only the dynein on select doublets are active in the beat cycle, which is necessary for the physical dictates of a flat beat, then there must be a mechanism to selectively inactivate the dyneins on the doublets that would contribute torque out of the beat plane. Selectively keeping the dyneins on Doublets #9, #1, #4 and #6 in the pre‐powerstroke bound state as seen in the Lin and Nicastro study (2018), but inactive, could be the mechanism that creates the ribbons responsible for establishment of a planar beat. The elaborate control system located on the central apparatus and the radial spokes could serve to regulate which dynein subsets are inactive. Ishijima et al. (1988) and (B. H. Gibbons et al., 1985) presented evidence that the CP plays a role in planar beating. Interaction of the CP apparatus with the spoke head proteins seems to be involved in regulation of the activation state of the dyneins associated with each doublet.

This may also be the key to understanding how flagella of *Chlamydomonas* and other algal flagellates can exhibit planar beating without the axonemal modifications found in the metazoa. The rotation of the CP in these organisms would appear to preclude a permanently defined principal beating plane. Nonetheless the beat in these organisms is still preferentially oriented in the plane defined by Doublets #1 and #5–6. Therefore, the ribbon hypothesis may be even more relevant to understanding how these flagella can define a beat plane and maintain a flat beat.

Free living flagellates are known to utilize their flagella for multiple functions. In order to allow for complex behaviors such as phototaxis, chemotaxis, mating and foraging, the rotating CP apparatus provides more diverse functionality to the flagella. The control of which doublets are inhibited and which can actively generate sliding in these organisms may rely on mechanical signaling conveyed via the rotation of the CP apparatus, as has been suggested by Oda, Yanagisawa, Yagi, and Kikkawa (2014).

There is another very puzzling experimental observation that seems to suggest that the beat plane can be shifted in any direction relative to the axoneme simply by initiating bending in a different plane at the base of the flagellum. Shingyoji and colleagues (Shingyoji, Gibbons, et al., 1991; Shingyoji, Katada, et al., 1991) demonstrated that imposed vibration could change the plane of the beat in sea urchin sperm. Taken at face value, this finding would suggest that the intact flagellum is capable of planar bending in any direction relative to the axoneme. However, drawing this conclusion is not justified by the limited information available. It has been observed that the head tail junction in sea urchin sperm is highly flexible (Brokaw, 1991; Sale, 1986). It is much more likely that the entire axoneme is forced to follow the imposed plane of vibration. It is possible that the axoneme is somewhat free to reorient relative to the head, either by rotating within the cell membrane or by twisting the head tail junction. The recoil through the same number of turns when the probe vibrations are discontinued would be consistent with this. If the axoneme was in the original orientation it would not need to recoil to resume its original beat plane. This explanation is much more likely than supposing that the axoneme can suddenly acquire a completely new mode of operation. That supposition defies the mechanical dynamics of the structures that normally restrict motion to a preferred plane. As we have no information about the orientation of the axoneme relative to the imposed vibrations, nothing further can really be concluded from this curious result.

### 2.1 The issue of torsion

Experimentally, the rigor wave studies of Gibbons and Gibbons (1974) demonstrated that sea urchin sperm flagella can exhibit torsion. When reactivated at low ATP and locked into rigor waves, the interbend regions seem to be stressed by sufficient torsion that each bend has a shifted plane.

We can make some interesting deductions concerning the potential for torsional distortion of the axoneme in flagella: (a) The force generated by dynein can generate torsional torque due to the fact that the outer doublets are centered ~80–90 nm from the central axis of the axoneme; (b) the magnitude of the torsional torque depends on the number of dynein contributing force to bend the axoneme and therefore increases with flagellar length; (c) all doublet pairs generate torsion in the same chirality, so the torsional torque is additive from off center doublets pairs on opposite sides of the axoneme. This is in contrast to the total bending torque, were doublet pairs on opposite sides of the axoneme oppose each other's contribution; (d) torsional resistance decreases with flagellar length, while bending resistance is relatively uniform. All of these factors make torsional distortion a larger factor in long flagella.

While it is possible to make these deductions from the principles of simple physics, it is presently not possible to quantitatively address the torsional contribution to flagellar mechanics. The total bending resistance of a dynein‐inhibited, simple 9 + 2 axoneme has been measured and is in the range of 6–9^e 8^ pN • nm ^2^ (Okuno, 1980; Okuno, Asai, Ogawa, & Brokaw, 1981; Pelle et al., 2009; Xu et al., 2016). In order to also incorporate the effects of torsion in modeling the axoneme in three dimensions it will be necessary to also know the torsional resistance, and that has not been measured.

Our rough torque calculation above for a short 20 μm flagellum, the torque for twisting is only about 10% of the bending torque. Consequently, torsion is likely a small factor for most cilia. However, it is likely to have a more significant role in a much longer flagella, because torsional resistance decreases with length and the total dynein force can also be greater.

Another consideration is the greater potential for two or more episodes of simultaneous activation (cycle bends) to be present in longer flagella. While activation of dyneins on microtubule doublets on the opposing side of the axoneme will bend the flagellum in the opposite direction, the torque contributing to torsion is oriented to twist the flagellum in the same direction (see Figure 2d). This may at least partially explain why very long flagella, such as found in insect sperm and fowl sperm, are often seen to maintain an overall spiral configuration.

Torsion may also provide some insight into the conversion of a planar beat into a helical beat at high mechanical and viscous loading as is observed in sea urchin and tunicate sperm (C. J. Brokaw, 1966, 1975; S. Ishijima, 2012; D. M. Woolley & Vernon, 2001). External mechanical resistance and viscous load have in common that both decrease the shear rate and consequently bring dynein closer to the stalling force limit. This will directly increase the tension on the doublets. In the geometric clutch model of flagellar beating, tension and compression on the doublets as a bend develops is the trigger for generating the transverse force that switches the dyneins “off.” At high viscosity, greater tension on the doublets will cause the “off” switching to occur at a reduced curvature (Lindemann, 1994a, 1994b). This will increase the potential for breaks in the transfer of torque across the entire axoneme, which we have shown is the requirement for planar beating.

Ishijima's analysis of the inter‐doublet shear in helical beating suggests that there is a sequential activation of doublet pairs (S. Ishijima, 2019). As we have illustrated in Figure 2d all doublet pairs contribute torsional torque in the same chiral direction. In a sequential helical activation of doublet pairs, each pair will contribute an off‐center bending torque which will have the effect of increasing the total accumulation of torsional torque. If the torque for bending is derived from pairs of active doublets rather than all the doublets on one side, this increases the development of off‐center torque derived from several independent doublet pairs.

Woolley and Vernon (2001) suggested the possibility that in helical beating doublet pairs may be activated on both sides of the flagellum simultaneously, much as we have illustrated in Figure 2d. This is an intriguing suggestion, as it would be the most extreme configuration for maximizing torsional torque. If this is indeed what is occurring in the helical beating mode, torsion may be sufficient to twist the flagellum so that the #3‐CP‐8 partition twists and aligns with the helical wave. Flagella of metazoan organisms such as sea urchin sperm have #5–6 bridges and a #3‐CP‐8 partition which make the flagellum stiffer in one plane. Therefore, even sequential activation of the doublets should not be capable of producing a true helical beat. However, if torsion is sufficient to twist the whole flagellum so that these stiffening structures are aligned perpendicular the bending waves, that might make a true helical configuration possible. This may explain how it is possible for flagella, like those of sea urchin sperm that have structures which increase bending resistance in one plane, to assume a truly helical beat. This possibility could be confirmed experimentally if the same cryo‐electron tomography technique successfully employed by Lin and Nicastro (2018), was utilized to examine a helically beating sea urchin sperm.

## CONFLICT OF INTEREST

The authors declare no conflicts of interest.

## Data Availability

Data sharing is not applicable to this article as no new data were created or analyzed in this study.
